# Signaling in Pollen Tube Growth: Beyond the Tip of the Polarity Iceberg

**DOI:** 10.3390/plants8060156

**Published:** 2019-06-07

**Authors:** Nolan Scheible, Andrew McCubbin

**Affiliations:** 1 School of Biological Sciences, Washington State University, Pullman, WA 99164-4236, USA; nolan.scheible@wsu.edu; 2 Center for Reproductive Biology, Pullman, WA, 99164, USA

**Keywords:** pollen tube tip growth, polarity, Ca^2+^ signaling, ROP GTPase

## Abstract

The coordinated growth of pollen tubes through floral tissues to deliver the sperm cells to the egg and facilitate fertilization is a highly regulated process critical to the Angiosperm life cycle. Studies suggest that the concerted action of a variety of signaling pathways underlies the rapid polarized tip growth exhibited by pollen tubes. Ca^2+^ and small GTPase-mediated pathways have emerged as major players in the regulation of pollen tube growth. Evidence suggests that these two signaling pathways not only integrate with one another but also with a variety of other important signaling events. As we continue to elucidate the mechanisms involved in pollen tube growth, there is a growing importance in taking a holistic approach to studying these pathways in order to truly understand how tip growth in pollen tubes is orchestrated and maintained. This review considers our current state of knowledge of Ca^2+^-mediated and GTPase signaling pathways in pollen tubes, how they may intersect with one another, and other signaling pathways involved. There will be a particular focus on recent reports that have extended our understanding in these areas.

## 1. Introduction

Pollen represents the male gametophyte of seed plants and as such plays the critical role of producing and transporting the sperm to facilitate fertilization in Gymnosperms and Angiosperms. Following deposition on the stigma by biotic or abiotic means, a compatible pollen grain will begin to germinate [1]. A tube protrudes from one of the apertures on the pollen wall, forming a conduit through which the sperm cells will travel to the egg [2]. The tube grows down through the style apoplast following a variety of chemotropic signals to the ovules [3]. Pollen tubes grow by rapid extension of the cell wall at the extreme apex of the elongating tube, commonly referred to as tip growth. This type of highly polarized cell growth can be observed among diverse but specialized eukaryotic cell types such as budding yeast and fungal hyphae, as well as other plant cells like root hairs [4]. Pollen tubes are some of the fastest growing plant cells and their growth requires tight coordination of a multitude of processes including actin dynamics, vesicle trafficking, ion fluxes, and signaling events [5]. The machinery that governs pollen tube growth acts to tightly regulate cell wall rheology by controlling wall integrity, deposition, recycling, and modification.

Rapidly extending the cell wall at a growing tip demands both the delivery and recycling of membrane and materials for cell wall synthesis at this growth point via exo- and endocytosis. Indeed, studies of vesicle trafficking and the actin dynamics involved have confirmed that these processes play a major role in tip growth, as treatment of pollen tubes with actin disrupting agents such as Latrunculin B, profilin, and Cytochalasin D inhibits growth [6]. The internal turgor pressure has been reported to be strictly regulated during pollen tube extension, but does not directly correlate with the growth rate, suggesting that regulation of wall extensibility is the controlling factor [7].

A pathway that plays a central role in coordinating polar growth in pollen tubes centers on plant Rho GTPase (termed ROP for Rho of Plants) activity. Coordinated ROP signaling is clearly important for tube growth as pollen overexpressing wildtype ROP, or expressing constitutively active or dominant negative ROP, exhibit perturbations of growth polarity resulting in swollen and sometimes almost spherical tube phenotypes [8]. ROP signal transduction has been linked to extracellular signals, two pollen specific transmembrane receptor kinases (PRKs) have been shown to interact with an *Arabidopsis* Rho Guanine Exchange Factor (RhoGEF) homolog from tomato in a phosphorylation dependent manner [9].

Calcium is a ubiquitous second messenger in plant cell signaling and its involvement in pollen tube growth has been the subject of extensive research over the last 20 years. A gradient of calcium has been found in the growing pollen tubes of more than 86 different species, demonstrating its conserved role in tip growth [10]. There is a steep calcium gradient from the apex of the tip back towards the shank in growing tubes, and its interruption by hyperosmotic conditions or the presence of calcium chelators results in elongation inhibition [11]. Calcium entry fluctuates at the tube apex and is correlated with pulses in growth rate [12]. The exact role of calcium in pollen tube growth is not well understood but it has been suggested that the influx causes an electrical gradient that affects the distribution (along the tube) of important membrane localized enzymes and/or that the pulsing Ca^2+^ gradient found at tube tips serves as an informational signal to activate calcium-sensing proteins [13]. Evidence supporting the latter has gained considerable support over the years with the identification and characterization of a spectrum of pollen-specific calcium sensing proteins involved in polar growth.

The substantial volume of data reported in recent years has dramatically improved our understanding of the signaling pathways governing polar growth in pollen tubes. Whilst we have amassed considerable knowledge about how pollen tube growth is regulated, particularly in regard to calcium and GTPase signaling, it is clear that this is the tip of the proverbial iceberg because lipid signaling, reactive oxygen species, and even metabolic events have all been implicated in this process. As will be discussed, available data suggests that tip growth in pollen tubes involves the action of an array of signaling events that overlap and exhibit considerable genetic and biochemical redundancy as well as multiple feedback loops. These act in concert to ensure successful fertilization and ensure a next generation of individuals. Extracellular signals from female reproductive tissues are perceived at the pollen tube tip by cell surface receptors, which transduce signals from the extracellular environment to the cytoplasmic interior of the pollen tube. Reception of these signals in the cytoplasm results in the activation of ROP GTPase to coordinate tube extension through a variety of downstream effectors including Ca^2+^. As tubes extend, ROP passes back from the extending tip into the membranes flanking the growing apex and are inactivated and sequestered in the cytoplasm to restrict ROP-stimulated growth to the pollen tube tip. Calcium acts as a spatial cue for the activity of a wide variety of calcium sensing proteins that help to coordinate growth regulation to ensure efficient polar growth.

This review focuses on the recent studies of two important signaling pathways involved in polar growth in pollen tubes, Ca^2+^-mediated and GTPase signaling pathways, in particular that there is evidence that they intersect with each other as well as signal molecules in other pathways. Proper functioning of these pathways is critical for efficient pollen tube growth, and the overlap between pathways to create a robust mechanism to coordinate this growth is likely to be essential to sexual reproduction in higher plants.

## 2. Calcium

### 2.1. Calcium Influx into Pollen Tubes

Calcium signaling in pollen tube growth has historically been the subject of many studies and remains of substantial focus today. Though a large volume of work has answered a considerable number of questions regarding the mechanisms of calcium signaling in plants, many remain. These include where the ion is stored, the identity of the channels responsible for calcium entry into cells, the gating and regulation of said channels, and perhaps most significantly, how cells differentially interpret different calcium signals like waves, gradients, and oscillations [14]. It has been widely assumed that calcium enters pollen tubes through one or more classes of ion channel and several have been implicated. Stretch-activated calcium channels have been identified in Lily pollen tubes [15]. Voltage-gated channels also contribute to calcium dynamics in pollen tubes and both calmodulin and actin dynamics could be involved in their regulation [16]. Hyperpolarization-activated calcium channels (HACCs) have been identified in pear and *Arabidopsis* using patch-clamp experiments and have shown to participate in Ca^2+^ dynamics in pollen [17,18]. Cyclic nucleotide gated channels (CNGC) have also been implicated. There are six *Arabidopsis* CNGCs specific to pollen, AtCNGC18 is necessary for proper tip growth in pollen tubes where it helps regulate calcium influx [19,20]. AtCNGC18 has been empirically shown to be a calcium-permeable membrane channel activated by cGMP and cAMP [21]. Regulation of AtCNGC18 is also mediated through phosphorylation by a calcium-dependent kinase, AtCPK32, to modulate calcium concentrations in the tube apex [22]. *AtCNGC18* is required for pollen tubes to reach the ovules to achieve fertilization, suggesting that it plays a role in guidance through the floral tissues [23]. However, two additional CNGCs, *AtCNGC7* and *8*, are also critical for the initiation of pollen tube growth as *AtCNGC7/AtCNGC8* double knockout lines have diminished transmission rates. Hence evidence supports the concerted actions of at least three *AtCNGCs* in effecting tube growth and guidance [24]. Much work has been done to tease apart mechanisms controlling calcium entry into pollen tubes and, excitingly, a model was recently proposed based on the identification of a calcium-based regulator of pollen CNGC channel activity, *AtCalmodulin2* (*AtCaM2*). AtCNGC8 forms a complex with AtCNGC18 and under low calcium concentrations, Ca^2+^-free CaM2 binds to this AtCNGC8/18 complex, opening a channel and allowing Ca^2+^ entry. When a critical Ca^2+^ concentration is reached, CaM2 binds the calcium causing its dissociation from the complex, inactivating the channel, and stopping Ca^2+^ influx [25]. Interestingly a root CNGC was recently identified and characterized in *Arabidopsis* (*AtCNGC14*), which, when expression levels are reduced, results in shortened root hairs and a disruption of the tip-focused calcium gradient [26], suggesting that CNGCs may be a conserved mechanism for calcium influx in tip growing plant cells. Another mode of calcium entry into pollen tubes is through the activity of glutamate receptor-like channels (GLRs). Twenty GLRs have been identified in *Arabidopsis*, six of which are expressed in pollen, and T-DNA insertion lines of two of these, *AtGLR1.2* and *3.7*, exhibit pollen tube growth defects, consistent with their playing a role in calcium influx [27]. These two GLRs were also shown to be activated by D-serine from pistil tissue indicating a role of GLRs in perceiving female guidance cues to navigate the tube through the floral apoplast [28]. Two more GLRs from *Arabidopsis*, AtGLR2.1, and 3.3 have also been found to be involved in pollen tip growth and their interaction with regulating/sorting proteins (CORNICHONs) is critical for establishing and maintaining appropriate calcium levels in growing pollen tubes [29]. As stated above, stretch-activated ion channels have also been implicated in the formation of the Ca^2+^ gradient in growing pollen tubes and one candidate family from *Arabidopsis*, MscS-like channels (MSLs), contains 10 members which are primarily thought to be membrane localized [30]. One of these members, MSL8, was shown to be involved in regulating osmotic forces associated with pollen germination and subsequent tube growth [31]. The role of MSL8 as a stretch-activated ion channel required for pollen function was confirmed with point-mutation lines that resulted in decreased germination and impaired tube integrity [32]. It is generally accepted that these classes of calcium channel are largely responsible for facilitating movement of calcium into plant cells, but the exact regulation of these channels and how they control the intricate temporal oscillations of the cation is still not well understood.

**Figure 1:**
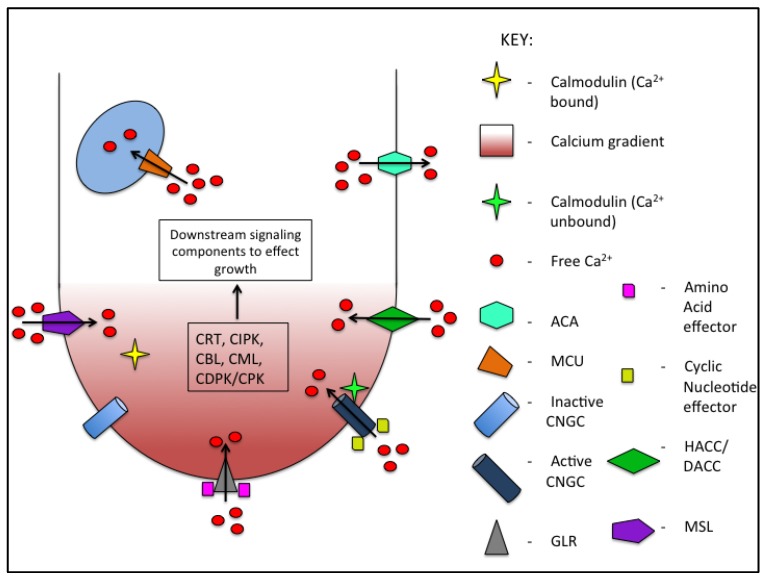
Overview of the actions of calcium signaling components. Calcium enters the cytoplasm of the tube through the activity of numerous membranous proteins including Cyclic nucleotide gated channels (CNGCs), Glutamate receptor-like channels (GLRs), Hyperpolarization/depolarization-activated calcium channels (HACC/DACCs), and MscS-like channels (MSLs). Calcium entry into the tube results in an elevation in calcium concentration. CNGCs and GLRs allow entry of calcium into the cytoplasm of the tube which causes an elevation in calcium concentration. CNGC is activated by calcium-free calmodulin. At critical calcium levels, calmodulin binds calcium causing its dissociation from the channel and the cessation of calcium entry. Calcium stimulates the activity of calcium-sensing proteins which result in calcium-mediated downstream signaling to affect growth. Mitochondrial calcium transporters (MCUs) in the mitochondrial membrane and autoinhibited Ca^2+^ ATPases (ACAs) at the plasma membrane facilitate the efflux of calcium out of the pollen tube, resulting in basal calcium levels.

As will be discussed below, calcium signaling is ubiquitous during pollen tube growth as it intertwines with other signaling cascades, therefore it is perhaps not surprising that so many different forms of calcium entry exist in pollen tubes. This redundancy in mechanisms of calcium entry renders investigations into how calcium gradients are maintained to be highly challenging. The cumulative results of these studies are summarized in Figure 1, to propose a general model for the regulation of calcium entry, sequestration, and signaling in pollen tubes.

### 2.2. Calcium Sequestration and Efflux from Pollen Tubes

The nature of fluctuating calcium concentrations in growing pollen tubes suggests that calcium sequestration and/or efflux are also under tight regulation. Calcium efflux transporters in plants have been more elusive than influx transporters. However, some efflux proteins have been identified in *Arabidopsis*, these are termed autoinhibited Ca^2+^ ATPases (ACAs) and there is a total of 10 isoforms. These are homologous to animal ACAs but differ in sub-cellular localization and in possessing regulatory sites at the N-terminal as opposed to the C-terminal ends [33]. One, *AtACA9*, which is expressed in pollen and root hairs, has been shown to be required for efficient tube growth as well as fertilization [34]. A recent report determined that AtACA9 physically interacts with a different calcium sensing protein, the copine AtBON1, and that this interaction is essential for germination and tube growth, highlighting the existence of a separate mode of pump regulation independent of calmodulin binding [35]. While characterization of *AtACA9* has provided a mode of exit for calcium, little else is known about how and where calcium is stored during oscillations, potential locations include the vacuole, endoplasmic reticulum, and mitochondria. It has been speculated that mitochondrial calcium dynamics could contribute to the overall calcium dynamics in pollen tube growth [36]. Indeed, a knockdown of expression of two mitochondrial calcium transporters, *AtMCU1* and *2*, was recently reported to cause pollen tube growth defects and reduced male transmission, supporting a role for mitochondrial calcium sequestration [37]. The apoplast, in particular the cell wall, has also been thought to act as a calcium sink in growing pollen tubes as calcium ions are a major constituent of cell wall materials, hence the cell wall could provide a destination for calcium efflux out of the symplast [38]. While these experiments provide evidence of some of the transporters involved in calcium movement in pollen (Figure 1), there is still much to be learned about the fate of intracellular calcium in growing pollen tubes.

### 2.3. Calcium Signaling—Deciphering the Code

The calcium code is highly complex and appears to be spatially and temporally specific, with waves of changing Ca^2+^ gradients resulting in different cellular responses. Calcium sensing proteins both decode and transmit signals intracellularly. One of the earliest studies implicating the involvement of such proteins in pollen tube growth, reported the identification of calcium-dependent phosphorylation activity in germinating pollen extracts [39]. Subsequently, a pollen specific calcium-dependent protein kinase was cloned from maize and shown to be required for pollen germination at a specific calcium concentration [40]. Calcium-dependent, or calmodulin-like domain, protein kinases (CDPKs/CPKs) have a high affinity for calcium and become biologically activated on binding it, whereby they phosphorylate specific downstream targets to transduce the calcium signal [41]. The function(s) of pollen specific CDPKs have been studied in several species. In petunia, overexpression of one (*PiCDPK1*) but not a second (*PiCDPK2*) was shown to result in pollen with the loss of polarity and growth arrest, as well as leading to a dramatic increase in cytosolic Ca^2+^ at the pollen tube tip (implying the existence of a positive feedback loop) [42]. Another CDPK/CPK from maize, *ZmCPK32*, is expressed in anthers and its kinase activity and sub-cellular localization are important for tube growth as modified inactive versions of the protein resulted in a reduction of the overexpression phenotype of reduced tube length [43].

The bulk of data on pollen CPKs comes from *Arabidopsis*, which possesses 34 CPK/CDPK isoforms [44]. Remarkably one fourth are expressed in pollen, suggesting that they are major players in receiving and transmitting the calcium code in growing pollen tubes [45]. A double knockout of two redundantly acting CDPK isoforms, *AtCPK17* and *AtCPK34*, (apparent orthologs of *PiCDPK1*) exhibits reduced pollen tube growth rates and is almost completely sterile [46]. These two isoforms plus an additional three, *AtCPK14*, *24*, and *32* all cause the loss of growth polarity in pollen tubes when over-expressed [44]. Furthermore, *AtCPK11* and *AtCPK24* have been shown to inhibit the activity of a K^+^ channel, shaker pollen inward K^+^ (SPIK) channel, through a phosphorylation cascade, thereby modulating cytoplasmic K^+^ concentrations to modulate growth [47]. Still, two more pollen *AtCPKs*, *2*, and *20*, have proven to be crucial for tip growth in pollen through activating an anion channel, a slow anion channel associated1 homolog (SLAH3), to maintain appropriate ion fluxes in growing tubes [48].

There is also evidence that calcineurin B-like interacting protein kinases (CIPKs) play a role in pollen tube tip growth. *Arabidopsis* *AtCIPK19*, has been reported to be involved in both the maintenance of the calcium gradient and cell polarity in tobacco pollen tubes [49]. In addition, AtCIPK12 has been shown to interact with calcineurin B-like proteins (CBL), AtCBL2, and 3 at the pollen vacuolar membrane to influence vacuole dynamics and overall tube extension [50]. Altering expression levels of these and two other pollen specific *Arabidopsis* CBLs, *AtCBL1*, and *9*, also causes defects in tube growth and morphology with phenotypes are exacerbated by altering ionic conditions [47,51].

Calmodulin-like (CML) proteins are also capable of transmitting calcium signals, few have been investigated for involvement in pollen tube growth but *AtCML24* from *Arabidopsis* has been reported to promote tube growth by affecting cytoskeletal dynamics [52]. Knock out mutants of another CML from *Arabidopsis*, *AtCML25*, display reduced sensitivity to exogenous Ca^2+^ along with elevated cytosolic calcium supporting a role for this calcium sensor in pollen tube growth [53]. Another class of calcium-sensitive protein, calreticulin (CRT), has been implicated in pollen tube growth in petunia. Knockdown of *PhCRT* expression in growing tubes alters the cytoskeletal actin dynamics as well as associated cytoplasmic streaming and the Ca^2+^ gradient, supporting a role for this protein in stabilizing ion fluxes coupled to the regulation of actin [54]. The function of *CRT* was previously investigated in tobacco pollen and hypothesized to play an important role in Ca^2+^ homeostasis [55]. The identification of pollen expressed proteins involved in interpreting the calcium code (Figure 1) has expanded our knowledge of how calcium signals help to coordinate tube growth. However, how pollen tubes coordinate differing calcium levels to specific physiological responses remains a major question.

### 2.4. Interaction Between Calcium Signaling and Other Signaling Pathways

Calcium has long been linked to important cell processes in tube growth such as endo- and exocytosis and cytoskeletal actin dynamics, but recent data suggest that many other signaling pathways and cascades interact with the tip focused calcium gradient. Boron is another nutrient essential for pollen tube growth, excess boron is toxic and causes deformed tubes, reduced growth rate and tube length, and also alters calcium gradients at the tube apex [56]. Conversely, boron deficiency also affects the calcium gradient, resulting in an increased Ca^2+^ influx, thus leading to an elevated cytosolic calcium concentration at the tube tip that also leads to changes in wall composition, specifically reducing the quantity of esterified pectins [57]. Nitric oxide (NO), another abundant signaling molecule in plants, also interacts with calcium signaling in pollen tubes. In pine pollen, both increased and decreased NO production perturb calcium gradients and alter actin dynamics, implying a functional link between NO signaling and calcium fluxes [58]. In RBOH (respiratory burst oxidase protein homolog), H and J both contain calcium-binding EF hand motifs and point mutations in these functional motifs affect reactive oxygen species (ROS) production in pollen tubes and also impaired complementation of the RBOH, H and J double-knockout phenotype [59]. The role of a polyamine spermine (spm) in pollen tube growth has also been investigated and exogenous treatment of tubes with 100 µm spm induced a calcium spike followed by altered tube growth [60].

Combined, these experiments suggest that calcium is an intricate signal in growing pollen tubes that integrates with other signaling mechanisms to coordinate tube growth. This calcium signal appears to be delicate and needs to be fine-tuned with intertwining pathways to be properly interpreted by the cell. This multifaceted nature of calcium signals is important to consider when investigating signal transduction pathways in pollen tubes. It would be informative to assess the effects a signaling pathway of interest might be having on the calcium gradient. Conversely, it would be prudent to consider secondary effects the calcium gradient might be causing. As calcium signals apparently interact with virtually all aspects of polar pollen tube growth, a holistic research framework is necessary to begin to truly unravel the calcium code and the messages it conveys.

## 3. GTPase Signaling

ROP GTPase signaling has been another area of substantial interest in pollen tube growth physiology as it evident that this pathway is a major player in the regulation of tube growth [61]. ROP activity acts as an important hub to convert signal perception into signal transduction by perceiving signals from cell surface receptors and through an effector domain propagating that signal to downstream components [62]. ROP signaling has been linked with calcium dynamics and several other key processes in tip growth, in particular vesicle trafficking and actin dynamics. Of the 11 ROP isoforms in the *Arabidopsis* genome, three have been shown to act redundantly to coordinate tip growth in pollen [63]. The activity of these ROP gene products is determined by a specialized set of regulatory proteins.

### 3.1. GTPase Regulatory Proteins

ROP regulation is mediated through the action of RhoGEFs, Rho GTPase activating proteins (RhoGAPs), and Rho guanine dissociation inhibitors (RhoGDIs) and the role of these regulatory proteins in pollen tip growth has been an area of interest. RhoGDIs are responsible for removing ROP from membranes and sequestering it in an inactive state in the cytosol, and studies in tobacco have demonstrated that a RhoGDI isoform is critical for spatially restricting ROP activity to the growing tube apex [63]. There are three RhoGDIs in *Arabidopsis*, *AtRhoGDI1*, *2a*, and *2b*, and triple-knockout plants of these genes show abnormal active ROP distribution in the membrane of tube tips and an apparent excess delivery of cell wall precursors to the tube apex leading to the loss of polar growth [64]. The RhoGEF function is critical in the ROP signaling a pathway to ensure appropriate biological activation (via the exchange of GDP for GTP) of ROPs during pollen tip growth [65,66]. As a result, targeting of RhoGEFs themselves is an important factor. Members of the AGC kinase family have been implicated in this process. AGC kinases are a diverse group of plant kinases and in *Arabidopsis* there are three expressed specifically in pollen: *AtAGC1.5*, *1.6*, and *1.7*. AtAGC1.5 has been experimentally shown to regulate RhoGEF targeting by the phosphorylation of a specific GEF domain to ensure accurate positioning of active ROP in the apical membrane of the pollen tube [67,68]. AGC kinases are regulated by 3-phosphoinositide-dependent protein kinase 1 (PDK1), which itself is regulated by signaling lipids such as phosphatidic acid, PtdIns3P, and PtdIns(3,4)P2 [69]. This work implies a link between lipid signaling and GTPase signaling pathways in tip growth.

**Figure 2:**
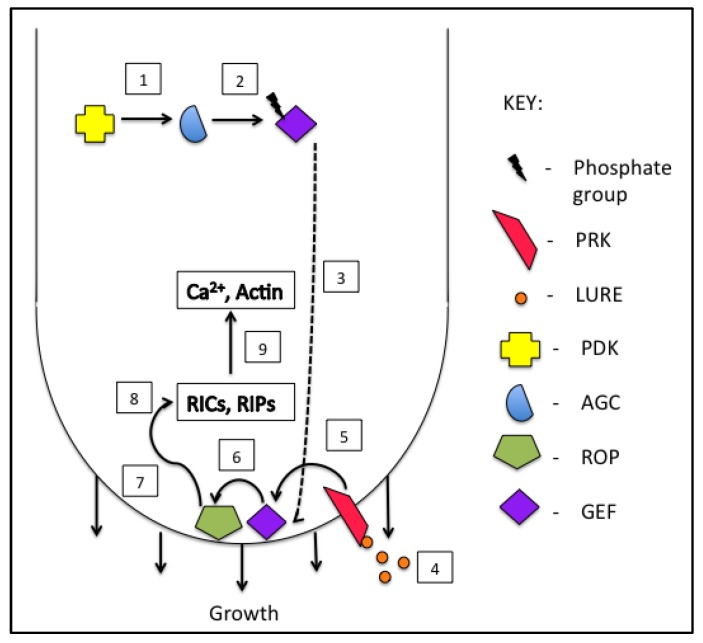
Overview of positive regulation of Rho GTPase (ROP). 3-phosphoinositide-dependent protein kinase (PDK) phosphorylates AGC kinase to regulate activity (1) which then goes on to phosphorylate Rho Guanine Exchange Factor (GEF) (2) affecting its localization to the membrane of the tube apex (3). Cysteine-rich polypeptide (LURE) molecules secreted from the transmitting tract stimulate activity of membrane localized pollen receptor-like kinase (PRK) (4), which activates GEF (5). GEF catalyzes the exchange of guanosine diphosphate (GDP) for guanosine triphosphate (GTP) and biological activation of ROP (6), which then activates downstream effectors such as ROP interacting CRIB-motif proteins (RICs) and ROP interactive partners (RIPs) (8). ROP effectors go on to modulate cell activities like Ca^2+^ gradients and actin dynamics (9) to affect growth.

Of the five *Arabidopsis* RhoGEFs expressed in pollen, two, *AtRhoGEFs 8* and *12*, interact with the kinase domain of a pollen receptor-like kinase (PRK), *AtPRK6*, and disturbance of this interaction results in pollen tubes with swollen tips and loss of polarity [70]. This study supports previous findings that established RhoGEFs as a link between PRK stimulation and ROP activity, and thus tube growth [71,72]. Additional support for the integration of PRK and ROP signaling during pollen tube growth comes from a study on two PRKs, BUPS (Buddha’s paper seal) 1 and 2, which determined that these kinases were required for normal growth through the transmitting tract and also that they interact with *AtRhoGEF1* and *12* in yeast 2-hybrid assays [73]. Figure 2 summarizes the above studies to provide a model for ROP activation in growing pollen tubes.

**Figure 3:**
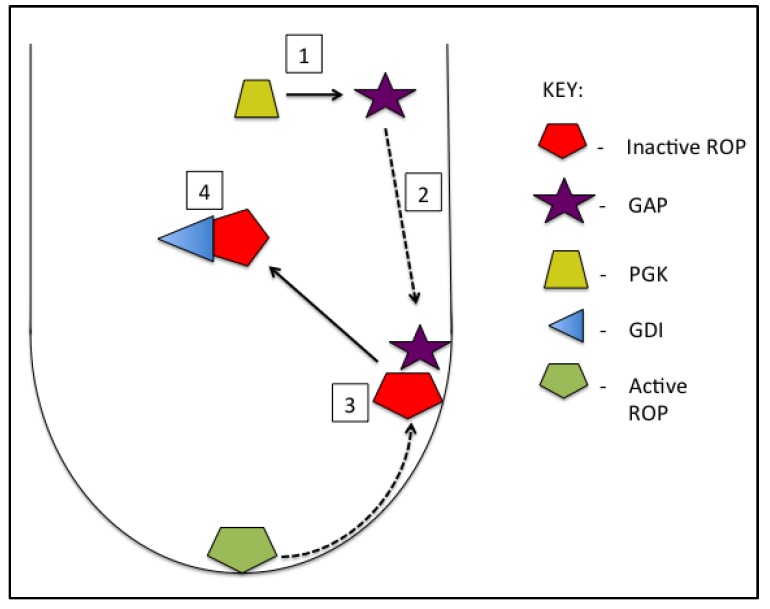
Overview of negative regulation of ROP GTPase. Phosphoglycerate kinase (PGK) acts on Rho GTPase activating proteins (GAPs) (1) to coordinate localization to the membranes flanking the tube apex (2). GAP stimulates the intrinsic GTPase activity of ROP, biologically inactivating the protein as it is passed along the growing tube membrane away from the apex (3). The biologically inactive ROP is bound to Rho guanine dissociation inhibitors (GDIs) in a protein complex sequestered in the cytosol (4).

RhoGAPs downregulate the biological activity of ROPs (by activating GTPase activity). The RhoGAP, *AtREN1* (*Rop1 Enhancer 1*), controls polarity in pollen tubes by spatially restricting ROP activity to the tube apex through a negative feedback mechanism [74] and more recently a second RhoGAP, *AtREN4*, was reported to also participate in this process [75]. Native localization of *AtREN1* to the membrane of the lateral region just behind the pollen tube tip, is abolished by overexpression of a phosphoglycerate kinase (PGK), leading to over-accumulation of active ROP at the tube tip and depolarized pollen tube growth [76]. This suggests a possible regulatory role for energy metabolism in the maintenance of ROP activity and tip growth. *AtREN1* activity is also influenced by the presence of salicylic acid (SA), a plant hormone commonly associated with defense signaling. Increased SA levels result in decreased GAP activity, and thus an increase in ROP activity, whereas methylated-SA is antagonistic to SA in this regard, suggesting the existence of yet another novel mechanism of tip growth regulation in pollen [77]. The role of RhoGAP activity in maintaining proper distribution of biologically active ROP in elongating tubes is consistent with studies of GAP characterization in tobacco that suggest GAP-mediated deactivation of ROP at the flanks of the tube apex [78]. Figure 3 summarizes the current knowledge of these negative regulators of ROP (and their own regulation) to provide a model for ROP inactivation in pollen tubes.

ROP signaling has also been studied in the context of pollen tube guidance through the floral tissues to the ovules. Combined experimental results and mathematical modeling have revealed a functional link between ROP-mediated exocytosis, tube sensing of guiding molecules, and the polarized growth of the tube towards the eggs [79]. *AtPRK6*, previously reported as interacting with *AtRhoGEF8* and *12*, was also implicated in the sensing of external chemical cues (LUREs) secreted by the female tissue that direct growth of the tube towards the micropylar end of the ovule. Excitingly, this establishes a mechanism for female influence on the growth of the male counterpart [80].

### 3.2. GTPase Signaling—Effectors

Downstream effectors of ROP signaling in pollen tubes have been studied to assess how these GTPases modulate tip growth. It is clear that ROPs act as a hub at the junction between signal perception and signal transduction and their activity has been implicated in the regulation of an array of cell structures and processes including microfilaments, microtubules, calcium levels, ROS production, lignin, and callose production, even gene expression [81]. A family of ROP interacting CRIB-motif (RIC) proteins have been identified as downstream targets of ROP activity. Nine members of this family are expressed in pollen and each cause growth effects when overexpressed in growing tubes [82]. Two, *AtRIC3* and *4*, play a role in the regulation of endo- and exocytosis via actin polymerization [83]. *AtRIC3* acts to mediate F-actin disassembly while *AtRIC4* acts antagonistically, promoting F-actin assembly, both through ROP activation [84]. Another member of the RIC protein family, *AtRIC1*, also plays a role in actin dynamics at the growing tube tip by severing microfilaments at the membrane and capping them in the cytoplasm at the tube apex [85]. RIC-like proteins have also been identified in lily and the function of one, LLPP12-2, was found to be similar to that of AtRIC3 [86] implying conservation of ROP target proteins across plant families. Other ROP effectors include a family of five proteins from *Arabidopsis* that each contain a conserved C-terminal region with the ability to facilitate interaction with ROP and hence termed ROP interactive partners (RIPs). One of these, *AtRIP1*, through overexpression and co-expression studies has been shown to participate in pollen tube ROP signaling through a positive feedback loop [87]. The same family of ROP effectors was also identified as interactors of constitutively active ROPs (ICRs) and functional analyses through knockdown and overexpression studies indicate they play a role in growth polarity in other plant cell types, such as epidermal pavement cells and root hairs [88]. ROP GTPases clearly play a complex role in the polarized growth of pollen tubes and intertwine with other signaling pathways as well as an array of downstream components. Clearly ROP GTPase activity represents another key signaling hub utilized by pollen tubes to coordinate polar growth. These molecular switches and their up and downstream components appear to be connected to not only calcium dynamics, but most, if not all, of the additional signaling pathways that underlie tip growth. Consequently, in addition to monitoring calcium levels, it is desirable to assess ROP GTPase activity when investigating individual pathways in the regulation of polar growth in pollen.

## 4. Conclusions

It is evident that there is tight regulation and concerted coordination of cell signaling activities during polar growth of pollen tubes. The considerable amount of work on tip growth in pollen has allowed us to compose a general picture of how this process is regulated. Extracellular signals from female reproductive tissues are perceived at the pollen tube tip by PRKs, which transduce signals from the extracellular environment to the cytoplasmic interior of the pollen tube. RhoGEFs receive these signals in the cytoplasm, being activated by the receptor-like kinases and then catalyze the activation of ROP GTPase to coordinate tube extension through a variety of downstream effectors including Ca^2+^. As the tube extends ROP, proteins pass back from the extending tip into the membranes flanking the actively growing apex and are inactivated by RhoGAPs and cytoplasmically sequestered by RhoGDIs to restrict ROP stimulated growth to the pollen tube tip. Calcium represents a spatial cue for the activity of a wide variety of calcium sensing proteins that help to coordinate growth activity and regulation, thus ensuring efficient polar growth. Through multiple feedback loops involving ROP, ionic channels, transporters, and their respective regulators, Ca^2+^ concentrations are tightly monitored to precisely modulate the downstream signals induced by the ion.

It is clear that two highly conserved signaling pathways, GTPase signaling and calcium signaling, intersect each other as well as with several other signaling pathways to effectively drive tip growth in pollen. The intersection of calcium-mediated and GTPase signaling pathways in the maintenance of polarized extension of pollen tubes represents an intricate and fine-tuned regulatory step in governing tip growth. Plants are far from the only organisms in which this intersection of common ubiquitous signaling pathways can be found. For example, Ca^2+^ influx is critical for the activation of a Rho GTPase involved in the development of human endothelial cells [89]. GTPase activity of a Sec14-like protein affects calcium release during embryogenesis in zebrafish and its depletion results in perturbed calcium signaling and developmental defects to the embryo [90] and free Ca^2+^ and a Rho GTPase are involved in a feedback loop in the development of colon cancer [91].

As humans, we have a tendency to try and compartmentalize what we know and assign it to strict groups and labels. There is a distinct danger that this kind of thinking is too narrow to allow us to unravel the complex interactions that underpin pollen tube growth. This complexity is perhaps not surprising, as the consequences of a failure of pollen tube growth would have dire consequences to biological fitness. Instead the evolution of pathways riddled with redundancy and alternate steps is a more logical scenario to ensure the ultimate delivery of sperm to the egg. The interplay between pathways remains largely unexplored and though studying the intersection of signaling pathways is complex and will be challenging, it will be essential to achieving a full understanding of the mechanisms underpinning pollen tube growth. While more versatile and robust methods need to be developed, there are tools currently that might be helpful in achieving this goal. Fluorescent probes, labels, and reporters are powerful ways to simultaneously image multiple aspects of cell dynamics including protein interactions and the cytoskeleton. Molecular sensors in the form of reporter proteins are constantly being developed and improved and recently two genetically encoded calcium optical imaging (GECO) proteins were used to image calcium in subcellular compartments, demonstrating the potential power of these techniques [92]. Imaging of active ROP GTPase is now possible using a truncated RIC4-green fluorescent protein (GFP) sensor [93]. Through the development of additional molecular sensors and the spectrally distinct fluorescent proteins now available, visualizing the effects of multiple pathways simultaneously is becoming possible. It will unquestionably be highly challenging to fill the remaining gaps in our knowledge but for the first time the ultimate goal of elucidating a fully integrated picture of signaling in pollen tube growth is becoming attainable.

## References

[1] Lord E.M. (2003). Adhesion and guidance in compatible pollination. J. Exp. Bot..

[2] Mascarenhas J.P. (1989). The male gametophyte of flowering plants. Plant Cell..

[3] Mizuta Y., Higashiyama T. (2018). Chemical signaling for pollen tube guidance at a glance. J. Cell Sci..

[4] Geitmann A., Emons A.M.C. (2000). The cytoskeleton in plant and fungal cell tip growth. J. Microsc..

[5] Franklin-Tong V.E. (1999). Signaling and the modulation of pollen tube growth. Plant Cell..

[6] Vidali L., McKenna S.T., Hepler P.K. (2001). Actin polymerization is essential for pollen tube growth. Mol. Biol. Cell..

[7] Benkert R., Obermeyer G., Bentrup F.-W. (1997). The turgor pressure of growing lily pollen tubes. Protoplasma..

[8] Li H., Lin Y., Heath R.M., Zhu M.X., Yang Z. (1999). Control of Pollen Tube Tip Growth by a Rop GTPase–Dependent Pathway That Leads to Tip-Localized Calcium Influx. Plant Cell.

[9] Kaothien P., Ok S.H., Shuai B., Wengier D., Cotter R., Kelley D., Kiriakopolos S., Muschietti J., McCormick S. (2005). Kinase partner protein interacts with the LePRK1 and LePRK2 receptor kinases and plays a role in polarized pollen tube growth. The Plant J..

[10] Brewbaker J.L., Kwack B.H. (1963). The essential role of calcium ion in pollen germination and pollen tube growth. Am. J. Bot..

[11] Pierson E.S., Miller D.D., Callaham D.A., Shipley A.M., Rivers B.A., Cresti M., Hepler P.K. (1994). Pollen tube growth 1s coupled to the extracellular calcium ion flux and the intracellular calcium gradient: Effect of BAPTA-type buffers and hypertonic media. Plant Cell.

[12] Pierson E.S., Miller D.D., Callaham D.A., van Aken J., Hackett G., Hepler P.K. (1996). Tip-localized calcium entry fluctuates during pollen tube growth. Dev. Biol..

[13] Feijo J.A., Malho R., Obermeyer G. (1995). Ion dynamics and its possible role during in vitro pollen germination and tube growth. Protoplasma..

[14] Feijo J.A., Wudick M.M. (2018). Calcium is life. J. Exp. Bot..

[15] Dutta R., Robinson K.R. (2004). Identification and characterization of stretch-activated ion channels in pollen protoplasts. Plant Physiol..

[16] Wang Y.-F., Fan L.-M., Zhang W.-Z., Zhang W., Wu W.-H. (2004). Ca^2+^-permeable channels in the plasma membrane of *Arabidopsis* pollen are regulated by actin microfilaments. Plant Physiol..

[17] Wu Y., Xu X., Li S., Liu T., Ma L., Shang Z. (2007). Heterotrimeric G-protein participation in *Arabidopsis* pollen germination through modulation of a plasma membrane hyperpolarization-activated Ca^2+^-permeable channel. New Phytol..

[18] Qu H.-Y., Shang Z.-L., Zhang S.-L., Liu L.-M., Wu J.-Y. (2007). Identification of hyperpolarization-activated calcium channels in apical pollen tubes of *Pyrus pyrifolia*. New Phytol..

[19] Shang Z.-L., Ma L.-G., Zhang H.-L., He R.-R., Wang X.-C., Cui S.-J., Sun D.-Y. (2005). Ca^2+^ influx into lily pollen grains through a hyperpolarization-activated Ca^2+^-permeable channel which can be regulated by extracellular CaM. Plant Cell Physiol..

[20] Chang F., Yan A., Zhao L.-N., Wu W.-H., Yang Z. (2007). A putative calcium-permeable cyclic nucleotide-gated channel, CNGC18, regulates polarized pollen tube growth. J. Integr. Plant Biol..

[21] Frietsch S., Wang Y.-F., Sladek C., Poulsen L.R., Romanowsky S.M., Schroeder J.I., Harper J.F. (2007). A cyclic nucleotide-gated channel is essential for polarized tip growth of pollen. Proc. Natl. Acad. Sci. USA.

[22] Gao Q.-F., Fei C.-F., Dong J.-Y., Gu L.-L., Wang Y.-F. (2014). *Arabidopsis* CNGC18 is a Ca^2+^-permeable channel. Mol. Plant.

[23] Zhou L., Lan W., Jiang Y., Fang W., Luan S. (2014). A calcium-dependent protein kinase interacts with and activates a calcium channel to regulate pollen tube growth. Mol. Plant.

[24] Gao Q.-F., Gu L.-L., Wang H.-Q., Fei C.-F., Fang X., Hussain J., Sun S.-J., Dong J.-Y., Liu H., Wang Y.-F. (2016). Cyclic nucleotide-gated channel 18 is an essential Ca^2+^ channel in pollen tube tips for pollen tube guidance to ovules in *Arabidopsis*. Proc. Natl. Acad. Sci. USA.

[25] Tunc-Ozdemir M., Rato C., Brown E., Rogers S., Mooneyham A., Frietsch S., Myers C.T., Poulsen L.R., Malho R., Harper J.F. (2013). Cyclic nucleotide gated channels 7 and 8 are essential for male reproductive fertility. PloS ONE.

[26] Pan Y., Chai X., Gao Q., Zhou L., Zhang S., Li L., Luan S. (2019). Dynamic interactions of plant CNGC subunits and calmodulins drive oscillatory Ca^2+^ channel activities. Dev. Cell..

[27] Zhang S., Pan Y., Tian W., Dong M., Zhu H., Luan S., Li L. (2017). *Arabidopsis* CNGC14 mediates calcium influx required for tip growth in root hairs. Mol. Plant.

[28] Michard E., Lima P.T., Borges F., Silva A.C., Portes M.T., Carvalho J.E., Gilliham M., Liu L.-H., Obermeyer G., Feijo J.A. (2011). Glutamate Receptor–Like genes form Ca^2+^ channels in pollen tubes and are regulated by pistil D-Serine. Science.

[29] Wudick M.M., Portes M.T., Michard E., Rosas-Santiago P., Lizzio M.A., Nunes C.O., Campos C., Damineli D.S.C., Carvalho J.C., Lima P.T. (2018). CORNICHON sorting and regulation of GLR channels underlie pollen tube Ca^2+^ homeostasis. Science.

[30] Basu D., Haswell E.S. (2017). Plant mechanosensitive ion channels: An ocean of possibilities. Curr. Opin. Plant Biol..

[31] Hamilton E.S., Jensen G.S., Maksaev G., Katims A., Sherp A.M., Haswell E.S. (2015). Mechanosensitive channel MSL8 regulates osmotic forces during pollen hydration and germination. Science.

[32] Hamilton E.S., Haswell E.S. (2017). The tension-sensitive ion transport activity of MSL8 is critical for its function in pollen hydration and germination. Plant Cell Physiol..

[33] Baxter I., Tchieu J., Sussman M.R., Boutry M., Palmgren M.G., Gribskov M., Harper J.F., Axelsen K.B. (2003). Genomic Comparison of P-Type ATPase Ion Pumps in *Arabidopsis* and Rice. Plant Physiol..

[34] Schiott M., Romanowsky S.M., Baekgaard L., Jakobsen M.K., Palmgren M.G., Harper J.F. (2004). A plant plasma membrane Ca^2+^ pump is required for normal pollen tube growth and fertilization. Proc. Natl. Acad. Sci. USA.

[35] Li Y., Guo J., Yang Z., Yang D.-L. (2018). Plasma membrane-localized calcium pumps and copines coordinately regulate pollen germination and fertility in *Arabidopsis*. Int. J. Mol. Sci..

[36] Colaco R., Moreno N., Feijo J.A. (2012). On the fast lane: Mitochondria structure, dynamics and function in growing pollen tubes. J. Microsc..

[37] Selles B., Michaud C., Xiong T.-C., Leblanc O., Ingouff M. (2018). *Arabidopsis* pollen tube germination and growth depend on the mitochondrial calcium uniporter complex. New Phytol..

[38] Holdaway-Clarke T.L., Feijo J.A., Hackett G.R., Kunkel J.G., Hepler P.K. (1999). Pollen tube growth and the intracellular cytosolic calcium gradient oscillate in phase while extracellular calcium influx is delayed. Plant Cell..

[39] Polya G.M., Micucci V., Rae A.L., Harris P.J., Clarke A.E. (1986). Ca^2+^-dependent protein phosphorylation in germinated pollen of *Nicotiana alata*, an ornamental tobacco. Physiol. Plant..

[40] Estruch J.J., Kadwell S., Merlin E., Crossland L. (1994). Cloning and characterization of a maize pollen-specific calcium-dependent calmodulin-independent protein kinase. Proc. Natl. Acad. Sci. USA.

[41] Harmon A.C., Gribskov M., Gubrium E., Harper J.F. (2001). The CDPK superfamily of.protein kinases. New Phytol..

[42] Yoon G.M., Dowd P.E., Gilroy S., McCubbin A.G. (2006). Calcium-Dependent Protein Kinase Isoforms in Petunia Have Distinct Functions in Pollen Tube Growth, Including Regulating Polarity. Plant Cell..

[43] Li J., Li Y., Deng Y., Chen P., Feng F., Chen W., Zhou X., Wang Y. (2018). A calcium-dependent protein kinase, ZmCPK32, specifically expressed in maize pollen to regulate pollen tube growth. PLoS ONE.

[44] Hrabak E.M., Chan C.W.M., Gribskov M., Harper J.F., Choi J.H., Halford N., Kudla J., Luan S., Nimmo H.G., Sussman M.R. (2003). The *Arabidopsis* CDPK-SnRK superfamily of protein kinases. Plant Physiol..

[45] Zhou L., Fu Y., Yang Z. (2009). A Genome-wide functional characterization of *Arabidopsis* regulatory calcium sensors in pollen tubes. J. Integr. Plant Biol..

[46] Myers C., Romanowsky S.M., Barron Y.D., Garg S., Azuse C.L., Curran A., Davis R.M., Hatton J., Harmon A.C., Harper J.F. (2009). Calcium-dependent protein kinases regulate polarized tip growth in pollen tubes. Plant J..

[47] Zhao L.-N., Shen L.-K., Zhang W.-Z., Zhang W., Wang Y., Wu W.-H. (2013). Ca^2+^-dependent protein kinase11 and 24 modulate the activity of the inward rectifying K^+^ channels in *Arabidopsis* pollen tubes. Plant Cell.

[48] Gutermuth T., Lassig R., POrtes M.-T., Maierhofer T., Romeis T., Borst J.-W., Hedrich R., Feijo J.A., Konrad K.R. (2013). Pollen tube growth regulation by free anions depends on the interaction between the anion channel SLAH3 and calcium-dependent protein kinases CPK2 and CPK20. Plant Cell.

[49] Zhou L., Lan W., Chen B., Fang W., Luan S. (2015). A Calcium sensor-regulated protein kinase, CALCINEURIN B-LIKE PROTEIN-INTERACTING PROTEIN KINASE19, is required for pollen tube growth and polarity. Plant Physiol..

[50] Steinhorst L., Mahs A., Ischebeck T., Zhang C., Zhang X., Arendt S., Schultke S., Heilmann I., Kudla J. (2015). Vacuolar CBL-CIPK12 Ca^2+^-sensor-kinase complexes are required for polarized pollen tube growth. Curr. Biol..

[51] Mahs A., Steinhorst L., Han J.-P., Shen L.-K., Wang Y., Kudla J. (2013). The Calcineurin B-Like Ca^2+^ Sensors CBL1 and CBL9 function in pollen germination and pollen tube growth in *Arabidopsis*. Mol. Plant.

[52] Yang X., Wang S.-S., Wang M., Qiao Z., Bao C.-C., Zhang W. (2014). *Arabidopsis thaliana* calmodulin-like protein CML24 regulates pollen tube growth by modulating the actin cytoskeleton and controlling the cytosolic Ca^2+^ concentration. Plant Mol. Biol..

[53] Wang S.-S., Diao W.-Z., Yang X., Qiao Z., Wang M., Acharya B.R., Zhang W. (2015). *Arabidopsis thaliana* CML25 mediates the Ca^2+^ regulation of K^+^ transmembrane trafficking during pollen germination and tube elongation. Plant Cell Environ..

[54] Suwinska A., Wasag P., Zakrzewski P., Lenartowska M., Lenartowski R. (2017). Calreticulin is required for calcium homeostasis and proper pollen tube tip growth in *Petunia*. Planta.

[55] Nardi M.C., Feron R., Navazio L., Mariani P., Pierson E., Wolters-Arts M., Knuiman B., Mariani C., Derksen J. (2006). Expression and localization of calreticulin in tobacco anthers and pollen tubes. Planta.

[56] Fang K., Zhang W., Xing Y., Zhang Q., Yang L., Cao Q., Qin L. (2016). Boron ‘*Malus domestica* pollen tube growth. Front. Plant Sci..

[57] Fang K.F., Du B.S., Zhang Q., Xing Y., Cao Q.Q., Qin L. (2019). Boron deficiency alters cytosolic Ca^2+^ concentration and affects the cell wall components of pollen tubes in *Malus domestica*. Plant Biol..

[58] Wang Y., Chen T., Zhang C., Hao H., Liu P., Zheng M., Baluska F., Samaj J., Lin J. (2009). Nitric oxide modulates the influx of extracellular Ca^2+^ and actin filament organization during cell wall construction in *Pinus bungeana* pollen tubes. New Phytol..

[59] Kaya H., Nakajima R., Iwano M., Kanaoka M.M., Kimura S., Takeda S., Kawarazaki T., Senzaki E., Hamamura Y., Higashiyama T. (2014). Ca^2+^-activated reactive oxygen species production by *Arabidopsis* RbohH and RbohJ is essential for proper pollen tube tip growth. Plant Cell.

[60] Aloisi I., Cai G., Faleri C., Navazio L., Serafini-Fracassnini D., Del Duca S. (2017). Spermine regulates pollen tube growth by modulating Ca^2+^-dependent actin organization and cell wall structure. Front. Plant Sci..

[61] Zheng Z.-L., Yang Z. (2000). The Rop GTPase: An emerging signaling switch in plants. Plant Mol. Biol..

[62] Yang Z. (2002). Small GTPases: Versatile signaling switches in plants. Plant Cell..

[63] Klahre U., Becker C., Schmitt A.C., Kost B. (2006). Nt-RhoGDI2 regulates Rac/Rop signaling and polar cell growth in tobacco pollen tubes. The Plant J..

[64] Feng Q.-N., Kang H., Song S.-J., Ge F.-R., Zhang Y.-L., Li E., Li S., Zhang Y. (2016). *Arabidopsis* RhoGDIs are critical for cellular homeostasis of pollen tubes. Plant Physiol..

[65] Berken A., Thomas C., Wittinghofer A. (2005). A new family of RhoGEFs activates the Rop molecular switch in plants. Nature.

[66] Gu Y., Li S., Lord E.M., Yang Z. (2006). Members of a novel class of *Arabidopsis* Rho guanine nucleotide exchange factors control Rho GTPase-dependent polar growth. Plant Cell.

[67] Zhang Y., He J., McCormick S. (2009). Two *Arabidopsis* AGC kinases are critical for the polarized growth of pollen tubes. Plant J..

[68] Li E., Cui Y., Ge F.-R., Chai S., Zhang W.-T., Feng Q.-N., Jiang L., Li S., Zhang Y. (2018). AGC1.5 kinase phosphorylates RopGEFs to control pollen tube growth. Mol. Plant.

[69] Bogre L., Okresz L., Henriques R., Anthony R.G. (2003). Growth signalling pathways in *Arabidopsis* and the AGC protein kinases. Trends Plant Sci..

[70] Yu Y., Song J., Tian X., Zhang H., Li L., Zhu H. (2018). *Arabidopsis* PRK6 interacts specifically with AtRopGEF8/12 and induces depolarized growth of pollen tubes when overexpressed. Sci. China Life Sci..

[71] Zhang Y., McCormick S. (2007). A distinct mechanism regulating a pollen-specific guanine nucleotide exchange factor for the small GTPase Rop in *Arabidopsis thaliana*. Proc. Natl. Acad. Sci. USA.

[72] Chang F., Gu Y., Ma H., Yang Z. (2013). AtPRK2 promotes ROP1 activation via RopGEFs in the control of polarized pollen tube growth. Mol. Plant.

[73] Zhu L., Chu L.-C., Liang Y., Zhang X.-Q., Chen L.-Q., Ye D. (2018). The *Arabidopsis* CrRLK1L protein kinases BUPS1 and BUPS2 are required for normal growth of pollen tubes in the pistil. Plant J..

[74] Hwang J.-U., Vernoud V., Szumlanski A., Nielsen E., Yang Z. (2008). A tip-localized Rho GTPase-activating protein controls cell polarity by globally inhibiting Rho GTPase at the cell apex. Curr. Biol..

[75] Li H., Luo N., Wang W., Liu Z., Chen J., Zhao L., Tan L., Wang C., Qin Y., Li C. (2018). The REN4 rheostat dynamically coordinates the apical and lateral domains of *Arabidopsis* pollen tubes. Nat. Comm..

[76] Chen W., Gong P., Guo J., Li H., Li R., Xing W., Yang Z., Guan Y. (2018). Glycolysis regulates pollen tube polarity via Rho GTPase signaling. PLoS Genet..

[77] Rong D., Luo N., Mollet J.C., Liu X., Yang Z. (2016). Salicylic acid regulates pollen tip growth through an NPR3/NPR4-independent pathway. Mol. Plant.

[78] Klahre U., Kost B. (2006). Tobacco RhoGTPase ACTIVATING PROTEIN1 spatially restricts signaling of RAC/Rop to the apex of pollen tubes. Plant Cell.

[79] Luo N., Yan A., Liu G., Guo J., Rong D., Kanaoka M.M., Xiao Z., Xu G., Higashiyama T., Cui X. (2017). Exocytosis-coordinated mechanisms for tip growth underlie pollen tube growth guidance. Nat. Comm..

[80] Takeuchi H., Higashiyama T. (2016). Tip-localized receptors control pollen tube growth and LURE sensing in *Arabidopsis*. Nature.

[81] Berken A. (2006). ROPs in the spotlight of plant signal transduction. Cell Mol. Life Sci..

[82] Wu G., Gu Y., Li S., Yang Z. (2001). A Genome-wide analysis of *Arabidopsis* Rop-Interactive CRIB motif–containing proteins that act as Rop GTPase targets. Plant Cell.

[83] Lee Y.J., Szumlanski A., Nielsen E., Yang Z. (2008). Rho-GTPase–dependent filamentous actin dynamics coordinate vesicle targeting and exocytosis during tip growth. J. Cell Biol..

[84] Gu Y., Fu Y., Dowd P., Li S., Vernoud V., Gilroy S., Yang Z. (2005). A Rho family GTPase controls actin dynamics and tip growth via two counteracting downstream pathways in pollen tubes. J. Cell Biol..

[85] Zhou Z., Shi H., Chen B., Zhang R., Huang S., Fu Y. (2015). *Arabidopsis* RIC1 severs actin filaments at the apex to regulate pollen tube growth. Plant Cell.

[86] Hsu S.-W., Wang C.-S. (2010). Lily Cdc42/Rac-interactive binding motif-containing protein, a Rop target, involves calcium influx and phosphoproteins during pollen germination and tube growth. Plant Signal. Behav..

[87] Li S., Gu Y., Yan A., Lord E., Yang Z.-B. (2008). RIP1 (ROP Interactive Partner 1)/ICR1 marks pollen germination sites and may act in the ROP1 pathway in the control of polarized pollen growth. Mol. Plant.

[88] Lavy M., Bloch D., Hazak O., Gutman I., Poraty L., Sorek N., Sternberg H., Yalovsky S. (2007). A novel ROP/RAC effector links cell polarity, root-meristem maintenance, and vesicle trafficking. Curr. Biol..

[89] Masiero L., Lapidos K.A., Ambudkar I., Kohn E.C. (1999). Regulation of the RhoA pathway in human endothelial cell spreading on type IV collagen: Role of calcium influx. J. Cell Sci..

[90] Gong B., Shen W., Xiao W., Meng Y., Meng A., Jia S. (2017). The Sec14-like phosphatidylinositol transfer proteins Sec14l3/SEC14L2 act as GTPase proteins to mediate Wnt/Ca^2+^ signaling. eLife.

[91] Stadler S., Nguyen C.H., Schachner H., Milovanovic D., Holzner S., Brenner S., Eichsteininger J., Stadler M., Senfter D., Krenn L. (2017). Colon cancer cell-derived 12(S)-HETE induces the retraction of cancer-associated fibroblast via MLC2, RHO/ROCK and Ca^2+^ signalling. Cell. Mol. Life Sci..

[92] Kelner A., Leitao N., Chabaud M., Charpentier M., de Carvalho-Niebel F. (2018). Dual color sensors for simultaneous analysis of calcium signal dynamics in the nuclear and cytoplasmic compartments of plant cells. Front. Plant Sci..

[93] Hwang J.-U., Gu Y., Lee Y.-J., Yang Z. (2005). Oscillatory ROP GTPase activation leads the oscillatory polarized growth of pollen tubes. Mol. Biol. Cell..

